# Editorial: Key players of the immune system in skin inflammation—allergy and autoimmunity

**DOI:** 10.3389/falgy.2024.1536289

**Published:** 2025-01-10

**Authors:** Laila Karra Khalil, Abhishek Asthana, Roopesh Singh Gangwar

**Affiliations:** ^1^ Cartherics Pty Ltd, Notting Hill, VIC, Australia; ^2^ Cancer Biology, Lerner Research Institute, Cleveland Clinic, Cleveland, OH, United States; ^3^ Division of Allergy and Clinical Immunology, School of Medicine, University of Virginia, Charlottesville, VA, United States

**Keywords:** skin inflammation, eosinophils, allergy, autoimmunity, mast cells, skin microbiome

**Editorial on the Research Topic**
Key players of the immune system in skin inflammation—allergy and autoimmunity

The immune system employs three lines of defense: (1) physical and physiological barriers, (2) innate immunity, and (3) adaptive immunity. The *Skin*, the largest organ of the human body, serves as the first line of defense by acting as a physical barrier. The skin immune system comprises resident cells, innate immune cells, and cells of myeloid and lymphoid origin. Together, these components provide robust protection across all three lines of defense. The skin hosts a diverse microbiome of symbiotic and pathogenic bacteria known as *The Skin Microbiome*. Dysregulated immune responses and bacterial imbalances (dysbiosis) can disrupt the host defense, resulting in impaired healing, tissue dysfunction, and inflammatory conditions. The skin is a site where sterile inflammation, including allergies and autoimmune conditions like atopic dermatitis (AD), contact dermatitis, eczema, vitiligo, lupus, psoriasis, and hidradenitis suppurativa, can occur.

A key aspect of skin immunity is the crosstalk among skin-resident cells, including mast cells, eosinophils, NK cells, neutrophils, Langerhans cells, macrophages, keratinocytes, T cells, and nerve cells. This interaction drives immune responses, which further evolve with the recruitment of cells of myeloid and lymphoid origin. For example, mast cells and eosinophils are central effector cells of immediate-type allergic inflammation, while T cells and their subsets dominate delayed type allergic inflammation and some autoimmune disorders (1, 2). Mast cells which are crucial for IgE-mediated allergic reactions, are tissue-resident cells while eosinophils are granulocytic leukocytes that are recruited to the inflamed tissues during inflammation. Both AD and psoriasis are common inflammatory skin diseases involving skin barrier damage and dysfunction. Inflammatory responses are characterized by infiltrating cell populations, cellular metabolism, and the release of cytokines, chemokines, and growth factors. Additionally, the skin microbiome plays a critical role in modulating immune responses.

This research topic is focused on providing immune mechanisms driving skin inflammation, to improve early diagnosis and advance safer, more effective treatments for allergic and autoimmune skin diseases.

Eosinophils play roles beyond allergy. Chronic inflammatory environments enable their long-term survival in tissues, leading to unresolved inflammation, fibrosis, and tissue remodeling. Novel therapeutic strategies are needed to resolve eosinophil-mediated inflammation. Recent studies have highlighted the non-antibiotic properties of tetracyclines (TCNs) in conditions like asthma and bullous pemphigoid (3). Gehring et al. demonstrated that TCNs induce mitochondria-mediated, caspase-dependent apoptosis in eosinophils, downregulate activation markers, and counter the pro-survival effects of cytokines like IL-3, IL-5, and GM-CSF (Gehring et al.). Unlike corticosteroids, which are effective but have side effects, TCNs could benefit steroid-unresponsive patients. Further research on repurposing FDA-approved drugs could yield additional therapeutic options.

AD, a systemic disease, is characterized by skin barrier dysfunction and elevated inflammatory cytokines in circulation. Though their role as markers of disease progression remains unclear. Using genome-wide association studies (GWAS) and patient samples, Xuan et al. have identified a panel of elevated cytokines (IL-13, IL-18R1, TNFSF14, and TRANCE) as potential risk factors for AD. Conversely, cytokines like TNF-β, CD5, CXCL11, and IL-33 were negatively associated with AD risk. The findings emphasize the importance of integrating genomic data from skin samples, not just serum, to unravel cytokine roles in AD. In AD lesional skin, monocytes-derived dendritic epidermal cells (IDECs) express the high-affinity IgE receptor (FcɛRI) and Toll-like receptor 2 (TLR2), however, how their cellular metabolism modulates inflammation remained unclear. Gao et al. demonstrated that IDEC activation via TLR2 (Pam3CSK4) or FcɛRI (anti-IgE) triggers glycolysis, enhances inflammatory marker expression, and promotes cytokine release. IDECs treated with Pam3CSK4, alone or combined with anti-IgE demonstrated upregulated inflammatory markers and cytokine production. These treatments stimulated glycolysis, resulting in increased lactate production without impairing mitochondrial function. These results suggest glycolysis as a metabolic driver of IDEC-mediated inflammation in AD.

*The skin microbiome* plays a critical role in the development and progression of AD and psoriasis (4). Skin colonization by *Staphylococcus aureus* is a hallmark of AD pathogenesis. Cell-specific gene knockout and transgenic mouse models have been valuable tools for unraveling gene function in specific cell types involved in AD and psoriasis (5–7). One such gene of interest is the signal transducer and activator of transcription 3 (STAT3), a transcription factor involved in numerous biological processes. Wang et al. utilized an epidermal keratinocyte-specific STAT3-deficient mouse (STAT*3 cKO* mice) to investigate the role of STAT3 in keratinocytes in AD. They demonstrated that topical application of 2,4-dinitrochlorobenzene (DNCB) exacerbated AD-like inflammation in these mice involving increased cell proliferation, reduced expression of filaggrin and loricrin, and downregulation of S100A9. Interestingly, the microbial population in the skin of STAT3 *cKO* mice shifted from *Ralstonia* to *Staphylococcus*, emphasizing the importance of STAT3 in maintaining skin barrier function and regulating the skin microbiome (Wang et al.).

Unlike AD, psoriasis is a complex chronic immune-mediated disease involving genetic, epigenetic, environmental and immune factors. Psoriasis is a Th17-mediated autoimmune skin inflammation primarily governed by Treg dysfunction and the IL-17 family of cytokines (IL-17, IL-22, IL-23, IL-6, and TNF-a) which play pivotal roles in psoriasis pathogenesis. Emerging evidence highlights the role of sirtuins (SIRTs), particularly SIRT3 in psoriasis pathogenesis (8). A decrease in SIRT3 expression was observed in macrophages of psoriasis patients and in mouse models of psoriasis (8, 9). However, the exact role of SIRT3 in psoriasis remained poorly understood. Guo et al. demonstrated that SIRT3 alleviates imiquimod-induced psoriatic skin inflammation through deacetylation of XBP1s and modulation of TLR7/8, inducing IL-23 production in macrophages. These results indicate that SIRT3 deficiency exacerbates psoriatic inflammation through increased acetylation of XBP1s, leading to heightened IL-23 production in macrophages (Guo et al.). These findings suggest novel insights into the anti-inflammatory role of SIRT3 and its therapeutic potential in psoriasis.

Taken together, the contributions to the Research Topic “*Key Players of the Immune System in Skin Inflammation—Allergy and Autoimmunity*” collectively shed light on critical immune mechanisms underlying skin inflammation. This compilation showcases examples ranging from drug repurposing and keratinocyte research in AD, to epigenetic regulation of psoriasis. Together, these studies advance our understanding of skin immunity and pave the way for innovative therapeutic approaches.
